# GluN2D-containing NMDA receptors-mediate synaptic currents in hippocampal interneurons and pyramidal cells in juvenile mice

**DOI:** 10.3389/fncel.2015.00095

**Published:** 2015-03-25

**Authors:** Jakob von Engelhardt, Christina Bocklisch, Lars Tönges, Anne Herb, Masayoshi Mishina, Hannah Monyer

**Affiliations:** ^1^Synaptic Signalling and Neurodegeneration, German Cancer Research Center (DKFZ)Heidelberg, Germany; ^2^Synaptic Signalling and Neurodegeneration, German Center for Neurodegenerative Diseases (DZNE)Bonn, Germany; ^3^Department of Clinical Neurobiology, Medical Faculty of Heidelberg University and DKFZHeidelberg, Germany; ^4^Brain Science Laboratory, The Research Organization of Science and Technology, Ritsumeikan UniversityKusatsu, Shiga, Japan

**Keywords:** NMDA receptors, hippocampus, interneurons, GluN2D, parvalbumin, development

## Abstract

The differential regulation of the two major *N*-methyl-D-aspartate receptor (NMDAR) subunits GluN2A and GluN2B during development in forebrain pyramidal cells has been thoroughly investigated. In contrast, much less is known about the role of GluN2D, which is expressed at low levels and is downregulated following the second postnatal week. However, it appears that few cells, presumably interneurons, continue to express GluN2D also in juvenile mice. To investigate which hippocampal cell types express this subunit, we generated transgenic mice with EGFP-tagged GluN2D receptors. The expression of the transgene was confined to hippocampal interneurons, most of which were parvalbumin- and/or somatostatin-positive. Electrophysiological and morphological analyses showed that GluN2D was present mainly in fast spiking basket and axo-axonic cells. Based on pharmacological evidence and electrophysiological analysis of GluN2D knockout mice, we conclude that GluN2D-containing NMDARs mediate synaptic currents in hippocampal interneurons of young and juvenile mice and in CA1 pyramidal neurons of newborn mice.

## Introduction

N-methyl-D-aspartate (NMDA) receptors are heteromers comprising two GluN1 and two GluN2 (Seeburg, 1993; Laube et al., 1998) or NR3 subunits (Ciabarra et al., 1995; Nishi et al., 2001). The composition of NMDARs influences functional properties such as kinetics, block by Mg^2+^-ions, agonist affinity and modulation by polyamines (Monyer et al., 1994; Sheng et al., 1994; Williams, 1995; Vicini et al., 1998). For example, recombinant receptors that contain GluN2A subunits display fast, GluN2B or GluN2C intermediate and GluN2D substantially slower decay kinetics (Monyer et al., 1994; Vicini et al., 1998). The four GluN2 subunits differ in their developmental and regional mRNA expression profile (Watanabe et al., 1992; Monyer et al., 1994). GluN2B and GluN2D are present during embryonic development, whereas GluN2A and GluN2C expression commences after birth. GluN2A and GluN2B display a strong expression in the cortex and hippocampus and GluN2C in the cerebellum of adult rodents. GluN2D expression is high during the first postnatal weeks especially in the diencephalon, brainstem (e.g., substantia nigra), cerebellum (deep cerebellar nuclei and Purkinje and Golgi cells) and spinal cord (Akazawa et al., 1994; Dunah et al., 1996, 1998; Cull-Candy et al., 1998; Jones and Gibb, 2005). In addition, a moderate GluN2D mRNA signal was observed in the cortex and hippocampus of young mice (Monyer et al., 1994). The distribution of GluN2D signal as described in this and another *in situ* hybridization study (Standaert et al., 1996) indicated that GluN2D-containing NMDARs are expressed mainly in interneurons of the hippocampus. However, single cell RT-PCR experiments and functional analysis indicated that GluN2D-containing NMDARs are also expressed by granule cells and pyramidal cells of young mice (Scherzer et al., 1998; Kirson et al., 1999; Hrabetova et al., 2000; Lozovaya et al., 2004; Harney et al., 2008).

Very little is known about the role of GluN2D-containing NMDARs in the hippocampus. Toward this goal, we first perform a detailed anatomical and functional analysis of GluN2D-containing neurons. Therefore, generated transgenic mice that express EGFP-tagged GluN2D receptors under the control of the GluN2D promoter (i.e., EGFP-GluN2D mice). Immunohistochemical analysis revealed that EGFP-GluN2D is expressed in hippocampal interneurons. Electrophysiological experiments using acute brain slices of EGFP-GluN2D mice and PV-EGFP mice (expression of EGFP under the control of the parvalbumin promoter) indicated that synaptic NMDAR-mediated excitatory postsynaptic currents (EPSCs) are partially mediated by GluN2D-containing NMDARs in hippocampal interneurons and pyramidal cells of young mice. The conclusions from these results were further substantiated by performing electrophysiological analysis of synaptic NMDAR-mediated EPSCs in hippocampal brain slices of mice with a genetic deletion of GluN2D (*grin2d^−/−^* mice) (Ikeda et al., 1995).

## Materials and methods

### Ethical approval

All experiments were approved by the Governmental Supervisory Panel on Animal Experiments of Baden-Wuerttemberg in Karlsruhe (T-86/10, A-22/11 and DKFZ237).

### Generation of EGFP-GluN2D transgenic mice

The screening of a mouse BAC library and selection of a suitable BAC was performed as described in Meyer et al. (2002). A 300 bp probe encompassing exons 1 and 2 of the mouse *grin2d* gene was generated by PCR. This probe was used to screen the mouse 129SV strain BAC library (Research Genetics, Inc., Huntsville, AL). Southern blot analysis of *NotI digested* BAC DNA separated by pulse-field gel electrophoresis (PFGE) analysis (CHEF-DRIII; Bio-Rad) was performed with a 430 bp PCR generated probe located in exon 1 of the *grin2d* gene to determine the size of the 5′- and 3′-flanking DNA. Of five BAC clones containing the *grin2d* gene, a clone with a genomic insert of 160 kb (at least 50 kb upstream and 30 kb downstream of the *grin2d* gene) was chosen for subsequent EGFP insertion via bacterial homologous recombination.

The targeting cassette comprised an artificial signal peptide sequence followed by the EGFP cassette and exon 1 of *grin2d* (lacking the signal peptide), and was flanked by two 500 bp homologous stretches of genomic DNA located upstream of the translational start and downstream of exon 1. The amplified 5′ and 3′ recombinogenic arms were cloned into pBluescript II SK (Stratagene). In a second step, the signal peptide-EGFP-exon 1 cassette was inserted between the two arms. The final recombination cassette was released via *Xhol* digestion and cloned into *Sal*I-digested pSV1recA to generate pSV1recA-EGFP-GluN2D. The method used for the integration of the EGFP-GluN2D cassette into the translational start of the *grin2d* gene of the BAC was as previously described (Yang et al., 1997).

BAC DNA was prepared by cesium chloride gradient centrifugation. After centrifugation and cutting the top of the tube, DNA was harvested with a 2 ml-wide bore plastic pipette to avoid shearing of the DNA. To release the BAC insert, 50 μg of BAC DNA was digested overnight with SrfI. A CL4B-Sepharose (Amersham Biosciences, Amersham Place, UK) column was equilibrated with 30 ml of injection buffer (in mM: 10 Tris-HCl, pH 7.5, 0.1 EDTA, and 100 NaCl) and was used to separate the released insert from the vector band. Aliquots of the collected 0.5 ml fractions were run on a PFGE gel to select the fractions used for subsequent pronuclear injection.

Isolated BAC insert was injected into pronuclei of B6D2F2 mouse zygotes at a concentration of 0.7 μg/ml. Founder animals were analyzed by PCR for the presence of EGFP with the following primers: EGFP-1 (CCACTAGTGTGAGCAAGGGCGAGGAGCT), EGFP-2 (GGACTAGTGCCGAGAGTGAT-CCCGGCGGCGGT). Two transgenic founder mice were bred with C57BL/6 mice. Transmission of the transgene was monitored in the offspring by PCR using EGFP-1 and EGFP-2 primers. In both lines, inheritance of the transgene followed Mendelian ratios. No changes in transgene expression pattern were observed between different generations.

### *In situ* hybridization

Brains were frozen on dry ice and cut into 12–16 μm sections on a microtome-cryostat. *In situ* hybridization experiments were carried out as described (Wisden and Morris, 1994) with two different antisense oligodeoxyribonucleotide probes (GluN2D oligo: 5′-CGTGGCCAGGCTTCGGTTATAGCCCACAGGACTGAGGT-3′; EGFP oligo: 5′-CACCATCTAATTCAACAAGAATTGGGACAACTCC-3′). The oligos were 3′ end-labeled by terminal deoxynucleotide transferase and (α)-^33^P-dATP (Hartmann Analytic, Germany). Brain sections were hybridized in 50% formamide, 4 × SSC (0.6 M NaCl, 0.06 M sodium citrate), 10% dextrane and 1 pg/μl labeled oligonucleotide at 42°C overnight and subsequently washed at 55°C for 30 min, dehydrated and exposed to Kodak® X-omat AR film for 1 week.

### Immunohistochemistry

Immunohistochemical studies were carried out on 50–75 μm free-floating coronal sections obtained from perfused brains of P3-5, P9-12, and adult EGFP-GluN2D mice (4% paraformaldehyde/0.1 M PBS, pH 7.4). The sections were washed with phosphate buffered saline (PBS) and permeabilized in PBS containing 0.4 % Triton X-100. Unspecific binding was blocked by adding 4% normal goat serum (NGS). Sections were incubated overnight at 4°C in PBS/0.1% Triton X-100/2%NGS. Double immunostaining experiments were performed with one primary antibody directed against EGFP (rabbit anti-GFP, 1:10,000, Invitrogen, Goettingen, Germany) to facilitate the detection of labeled cells and another primary antibody against interneuron marker proteins: mouse anti-calretinin, 1:5000 (Swant); mouse anti-calbindin, 1:5000 (Swant); mouse anti-parvalbumin, 1:3000 (Sigma-Aldrich, St.Louis, MO, USA), rat anti-somatostatin, 1:1000 (Chemicon, Temecula, USA); For visualization of primary antibodies, slices were incubated in PBS/1.5%NGS for 2.5 h at room temperature with the following secondary antibodies: goat anti-rabbit Alexa 488, 1:1000 (Invitrogen, Goettingen, Germany); goat anti-mouse Cy-3, 1:1000 (Jackson Immuno Research Laboratories Inc.,West Grove, PA); goat anti-rat Cy3, 1:1000 (Jackson Immuno Research Laboratories Inc.). Slices were subsequently mounted in Moviol onto microscope slides and analyzed using a bright field illumination fluorescent microscope (BX51W, Olympus, Japan) or a confocal microscope Zeiss LSM510 (Carl Zeiss, Germany).

### Electrophysiology

Two hundred and fifty micrometer-thick horizontal slices were prepared from brains of P3-P25 old mice. Slices were continuously superfused with ACSF (22–24°C) containing (in mM): 125 NaCl, 2.5 KCl, 2 CaCl_2_, 1 MgCl_2_, 1.25 NaH_2_PO_4_, 25 NaHCO_3_, and 25 glucose (pH 7.2, maintained by continuous bubbling with carbogen). Whole-cell recordings in current and voltage clamp mode (no liquid junction potential correction) were performed using pipettes with a resistance of 3–5 MΩ when filled with (in mM): 105 Cs-gluconate, 30 CsCl, 4 Mg-ATP, 10 phosphocreatine-Na, 0.3 GTP, and 10 HEPES, pH 7.3 (for recording of NMDAR-mediated currents) or when filled with 105 K gluconate, 30 KCl, 4 Mg-ATP, 10 phosphocreatine-Na, 0.3 GTP, and 10 HEPES, pH 7.3 (for recording of firing pattern of EGFP-GluN2D-positive cells). NMDAR-mediated EPSCs were evoked by Schaffer collateral/commissural fiber stimulation with a glass electrode filled with ACSF and were recorded at a holding potential of +40 mV in the presence of 10 μM of the NMDAR co-agonist glycine. NMDAR-mediated EPSCs were pharmacologically isolated with 10 μM SR95531 hydrobromide (gabazine, Biotrend, Wangen, Switzerland) and 10 μM 6-cyano-7-nitroquinoxaline-2,3-dione (CNQX, Biotrend) to block GABA_A_ and AMPA receptors, respectively. Ifenprodil hemitartrate (10 μM, Tocris) was used as a GluN2B subunit specific antagonist. Quantification of the amplitude and deactivation time constant was performed on averages of 100 NMDAR-mediated EPSCs. EPSC decays were fitted with two exponentials, a weighted tau was calculated according to: τ_decay_ = (τ_f_ × a_f_) + (τ_s_ × a_s_), where a_f_ and a_s_ are the relative amplitudes of the fast (τx_f_) and slow (τ_s_) exponential components. Access resistance was continuously monitored and data discarded if the resistance changed more than 20%.

The analysis of electrophysiological properties of EGFP-GluN2D-positive cells was performed essentially as described previously (von Engelhardt et al., 2011). Hyper- and depolarizing current pulses (1 s) were applied to calculate input resistance and threshold potential. Action potential waveform was analyzed at just suprathreshold potential. The action potential and afterhyperpolarization amplitude was measured from threshold to peak of the AP or AHP. The duration of the AP was measured at half amplitude. Maximal firing frequency was measured at the submaximal current step applied before spike inactivation became evident.

EGFP-positive neurons were visually identified using an upright microscope (BX51W1, Olympus, Japan) equipped with IR-DIC and standard epifluorescence. Stimulus delivery and data acquisition were performed using Patchmaster software (Heka Elektronik, Lambrecht, Germany). Signals were filtered at 3 kHz, sampled at 10 kHz and off-line analysis was performed using Igor Pro (Wavemetrics, Lake Oswego, OR, USA).

### Development of biocytin-filled cells

Brain slices of P3-25 old EGFP-GluN2D mice were prepared as described for electrophysiological experiments. Neurons were patch-clamped for 10–20 min with 0.5–1% biocytin in the pipette. After pipette withdrawal, slices were kept in the recording chamber for 10–20 min and subsequently immersion-fixed in 4% paraformaldehyde/0.1 M PBS for 8–16 h at 4°C. The slices were incubated with peroxidase-avidin-biotin complex (ABC, Vector Lab. Inc. Burlingame, CA, USA) and visualized using diaminobenzidine (DAB) chromogen. Slices were mounted onto microscope slides and coverslipped with Moviol. The biocytin-filled neuron reconstruction was performed using a Neurolucida 3D reconstruction system and the NeuroExplorer Software package (MicroBrightField, Colchester, VT, USA, and NeuroExplorer, Littleton, MA, USA).

### Statistics

Data are presented as mean ± standard deviation (SD) or median ± interquartile range [IQR]. Statistical differences between groups were examined by Mann–Whitney *U*-test for non-Gaussian distributed values. Normality of data distribution was tested by Kolmogorov–Smirnov test and equal variance by Bartlett's test. Statistical analysis was performed using the GraphPad Prism version 5.00 for Mac OS X (GraphPad Software, San Diego, CA, USA). *P*-values < 0.05 were considered statistically significant (^*^p < 0.05, ^**^p < 0.01, ^***^p < 0.001).

## Results

### EGFP-GluN2D is expressed in hippocampal interneurons

To identify GluN2D-expressing neurons in acute brain slices, we generated BAC transgenic mice that express the fusion protein EGFP and GluN2D (EGFP-GluN2D) under the control of the GluN2D promoter (Figure S1). Electrophysiological analysis of recombinant receptors comprising GluN1 and EGFP-GluN2D expressed in HEK293 cells provided evidence that the fusion of EGFP to the N-terminus of GluN2D does not interfere with the function of the receptor. EGFP-GluN2D/GluN1 receptors exhibit slow deactivation kinetics, which are characteristic for GluN2D-containing NMDARs (data not shown). *In situ* hybridization experiments with probes against EGFP and GluN2D on sagittal brain slices of postnatal day 14 (P14) and P21 old mice showed that the EGFP-GluN2D mRNA expression profile in EGFP-GluN2D-BAC mice (probe against EGFP) corresponds with the profile of endogenous GluN2D mRNA in wildtype mice (Figure S2). The expression was particularly high in the brain stem and the thalamus. Moderate expression was seen in cortex, hippocampus, olfactory bulb, and cerebellum. The GluN2D mRNA signal intensity was higher especially in thalamus, brainstem and olfactory bulb of EGFP-GluN2D-BAC mice when compared with the signal intensity in wildtype mice, indicating that as expected the expression of EGFP-GluN2D in BAC transgenic mice results in an overexpression of this NMDAR subunit.

**Figure 1:**
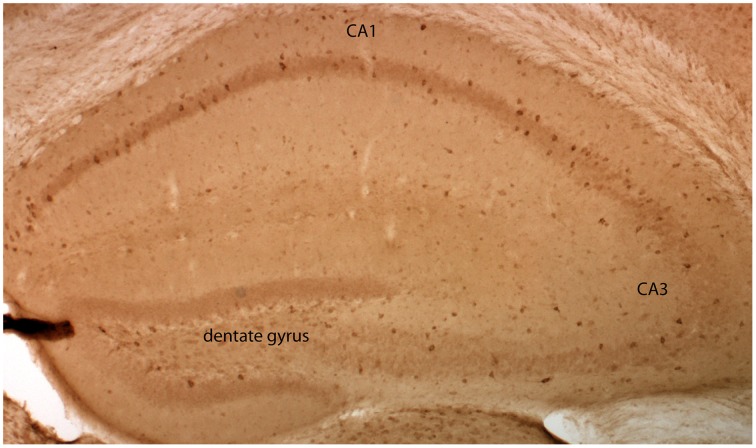
**GluN2D-EGFP is expressed in the hippocampus**. Visualization of EGFP-GluN2D in the hippocampus with an anti-EGFP antibody and DAB chromogen. EGFP-GluN2D-positive cells are localized in the CA1, CA2, dentate gyrus, and CA3 subregion. Most positive cells are close to or within the principal cell layer.

**Figure 2:**
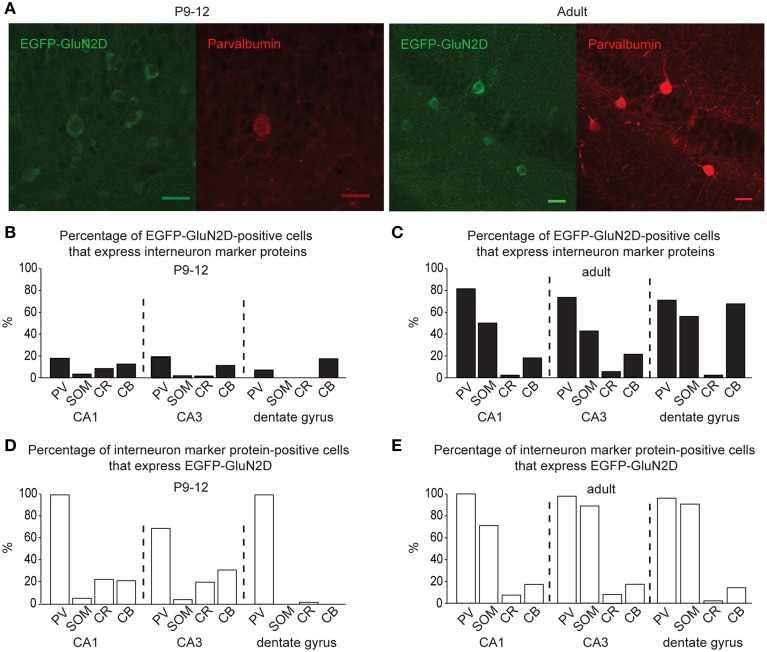
**GluN2D-EGFP is expressed in hippocampal interneurons. (A)** Immunohistochemical images of hippocampal EGFP-GluN2D-positive cells co-expressing PV in brains of P12 and adult mice. Scale bars = 30μm. **(B,C)** Quantification of EGFP-GluN2D-positive cells that express with PV, SOM, CR and CB in CA1, CA3 and dentate gyrus in P9-12 **(B)** and adult mice **(C)**, respectively. **(D,E)** Quantification of the percentage of interneuron marker-positive cells that are EGFP-GluN2D-positive in CA1, CA3, and dentate gyrus in P9-12 **(D)** and adult mice **(E)**, respectively.

To ease the interpretation of subsequent electrophysiological data, we performed an immunocytochemical based expression analysis at the cellular level. Immunohistochemistry with a primary antibody against EGFP revealed that EGFP-GluN2D is expressed in hippocampal cells. We inferred that most of these cells were interneurons, as their cell body was located in the hilus of the dentate gyrus, the stratum oriens, and stratum radiatum of the CA3, CA2, and CA1 area (Figure 1). There were many EGFP-GluN2D-positive cells also in the CA3, CA2, and CA1 principal cell layer. These might be either pyramidal cells or interneurons located in the principal cell layer, e.g., parvalbumin (PV)-positive cells (Celio and Heizmann, 1981). We investigated which cell types express EGFP-GluN2D by double-immunohistochemistry with antibodies against EGFP and interneuron marker proteins. Since GluN2D mRNA expression decreases dramatically with development in all brain areas, including the hippocampus, we performed immunohistochemistry (and all subsequent analyses) with brains from mice of three different age groups. Co-localization of EGFP-GluN2D with interneuronal marker proteins PV, somatostatin (SOM), calretinin (CR), and calbindin (CB) was investigated in hippocampi of P3-5, P9-12, and adult mice. EGFP-GluN2D-expressing cells were found in the hippocampus of mice of all three ages. Interneuron marker proteins, however, were barely detectable in neurons of P3-5 mice, rendering a quantification of co-localization useless. PV immunoreactivity was absent in P3-5, very faint in P9-12 and strong in adult mouse hippocampi (Figure 2A), reflecting a developmental upregulation of this protein consistent with previous observations (Nitsch et al., 1990). There was an increase in the proportion of interneuronal marker protein-expressing EGFP-GluN2D-positive cells from about forty percent in hippocampi of P9-12 mice to 100% in hippocampi of adult mice (Figures 2B,C). Since many EGFP-GluN2D-positive but marker protein-negative cells in P9-12 mice had a typical interneuron location (e.g., stratum oriens or radiatum), we assume that most, if not all, are indeed interneurons in which the developmental upregulation of interneuron marker proteins had not yet occurred. The profile of interneuron marker proteins that was detected in EGFP-GluN2D-positive cells was similar in the CA1, CA3, and dentate gyrus subregion in the hippocampus of adult mice: most EGFP-GluN2D positive neurons expressed either PV or SOM and to a lesser extent CB. Some fluorescent cells also expressed CR. One can infer that there is co-expression of PV and SOM in EGFP-GluN2D-positive neurons, given that PV and SOM are expressed in ca. 80 and 50% of fluorescent cells, respectively (Figure 2C).

EGFP-GluN2D is expressed in a substantial proportion of PV-, SOM-, CR-, and CB-positive interneurons. In fact, all PV-positive cells were also EGFP-GluN2D-positive in the CA1 area and dentate gyrus of P9-12 and adult mice. The percentage of SOM-positive cells that express EGFP-GluN2D increased with development. CR- and CB-positive interneurons expressed EGFP-GluN2D less frequently in the three hippocampal subregions (Figures 2D,E).

In summary, EGFP-GluN2D is expressed by many hippocampal interneurons, most of which co-express either PV or SOM in adult mice. Since all EGFP-GluN2D-positive cells expressed at least one of the four tested interneuronal marker proteins, we conclude that principal cells do not express EGFP-GluN2D or that the expression level is too low to be detectable.

### Physiological properties of EGFP-GluN2D-positive neurons

The expression of the fluorescent tag allowed us to detect EGFP-GluN2D-positive cells in acute slices and to investigate their physiological characteristics. However, overall the signal of EGFP-GluN2D-positive cells was very low. Thus, not all EGFP-GluN2D-positive cells could be identified in acute brain slices (the number of EGFP-GluN2D-positive cells was higher in fixed slices where the EGFP signal was enhanced upon anti-EGFP immunostaining). We focused on neurons in the CA1 area of the hippocampus for the analysis of functional and anatomical properties of GluN2D-positive cells. Action potential firing was evoked in the current-clamp mode by current injection. All EGFP-GluN2D-positive cells exhibited firing patterns that were typical for interneurons. Two cells in brain slices of P3-5 mice were too immature to respond with more than one small action potential. The electrophysiological properties of EGFP-GluN2D-positive cells showed developmental changes consistent with the maturation of hippocampal interneurons (see Table 1 for physiological properties). In particular, there was a four-fold increase in the maximal firing frequency and a five-fold decrease in the width of action potentials at half-height when comparing EGFP-GluN2D-positive cells in P3-5 mice with those in P20-25 mice. Seven out of 8 cells in brain slices of P20-25 mice exhibited firing patterns typical for fast spiking interneurons with a mean maximal firing frequency of 145 Hz, little frequency adaptation, high-amplitude and fast action potentials with little amplitude accommodation, deep and fast spike afterhyperpolarization (AHP) and an absence of afterdepolarization (ADP) or sag. One neuron in P20-25 mice exhibited a firing pattern with pronounced frequency adaptation at threshold potential, maximal firing frequency of 61 Hz, slow AHP and presence of a sag. Thus, most but not all EGFP-GluN2D-positive cells in P20-25 mice are fast-spiking interneurons. A clear-cut classification of EGFP-GluN2D-positive cells in P3-5 and P9-12 mice is not always possible as the firing pattern typical of mature interneurons is not fully established at this age. Nevertheless, the firing pattern of 9 out of 25 EGFP-GluN2D-positive cells of P9-12 mice resembled that of fast spiking interneurons in older mice (albeit with lower maximal firing frequency of 73 Hz), suggesting that those cells were immature fast-spiking interneurons.

**Table 1:** **Electrophysiological properties of EGFP-GluN2D-positive cells**.

	**P3-5**	**P9-11**	**>P21**
Input resistance (MΩ)	911 ± 692	296 ± 168	131 ± 33
Membrane potential (mV)	56 ± 9	65 ± 6	63 ± 8
Threshold potential (mV)	37 ± 2	41 ± 5	43 ± 5
Sag (mV)	3.4 ± 2.4	2.0 ± 2.7	0.86 ± 2.4
Amplitude of 1st AP (mV)	56 ± 11	76 ± 11	95 ± 10
Amplitude of 2nd AP (mV)	54 ± 11	74 ± 11	94 ± 11
1st AP half width (ms)	2.00 ± 0.53	1.00 ± 0.45	0.39 ± 0.12
2nd AP half width (ms)	2.25 ± 0.70	1.04 ± 0.47	0.41 ± 0.16
Amplitude of 1st AHP (mV)	13 ± 4	16 ± 5	23 ± 4
Amplitude of 2nd AHP (mV)	13 ± 4	17 ± 4	24 ± 3
Maximal frequency (Hz)	32.4 ± 16.5	54 ± 19	135 ± 47

### Anatomical features of EGFP-GluN2D-positive neurons

**Figure 3:**
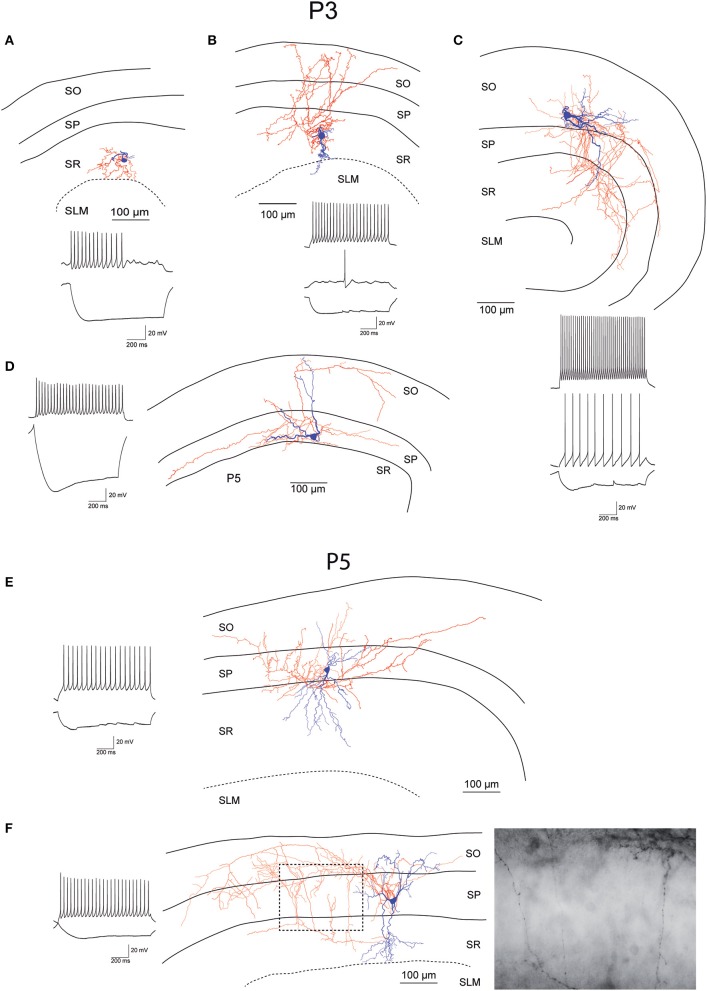
**Morphology and firing pattern of GluN2D-EGFP-positive cells in P3-5 transgenic mice. (A–D)** Reconstruction of four EGFP-GluN2D-positive cells (P3) with voltage responses to hyperpolarizing and depolarizing current injections. **(E,F)** Reconstruction of two EGFP-GluN2D-positive cells (P5) with voltage responses to hyperpolarizing and depolarizing current injections. Localization of cell body, dendritic and axonal arbors indicate that the two neurons may be an immature basket (upper) and an immature axo-axonic cell. The image of part of the axon in the stratum pyramidale of the lower cell (see dashed box in reconstruction) shows vertical rows of boutons (parallel to axon initial segments), typical for axo-axonic cells. SO, Stratum oriens; SP, stratum pyramidale; SR, stratum radiatum; SLM, stratum lacunosum-moleculare.

**Figure 4:**
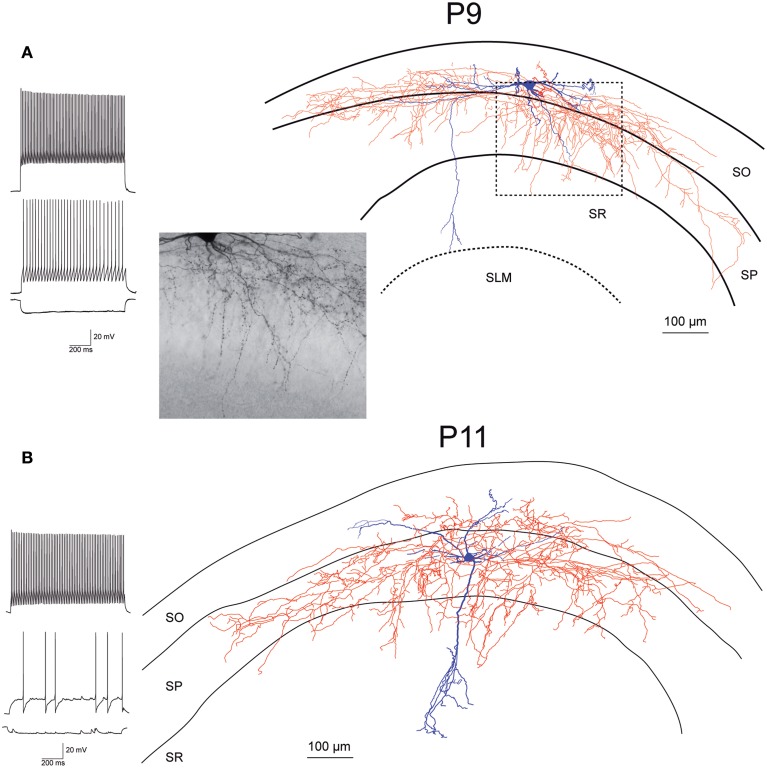
**Morphology and firing pattern of GluN2D-EGFP-positive cells in P9-11 transgenic mice. (A)** Reconstruction of an EGFP-GluN2D-positive cell (P9) with voltage responses to hyperpolarizing and depolarizing current injections. Firing pattern, localization of cell body, dendritic, and especially the axonal arbor at the border of SO and SP indicate that this is a fast-spiking axo-axonic cell. The image of part of the axon in the stratum pyramidale (see dashed box in reconstruction) shows vertical rows of boutons (parallel to axon initial segments), typical for axo-axonic cells. **(B)** Reconstruction of an EGFP-GluN2D-positive cell (P11) with voltage responses to hyperpolarizing and depolarizing current injections. Firing pattern and morphology (e.g., axonal arbor in SP) indicate that this is a fast-spiking basket cell. SO, Stratum oriens; SP, stratum pyramidale; SR, stratum radiatum; SLM, stratum lacunosum-moleculare.

**Figure 5:**
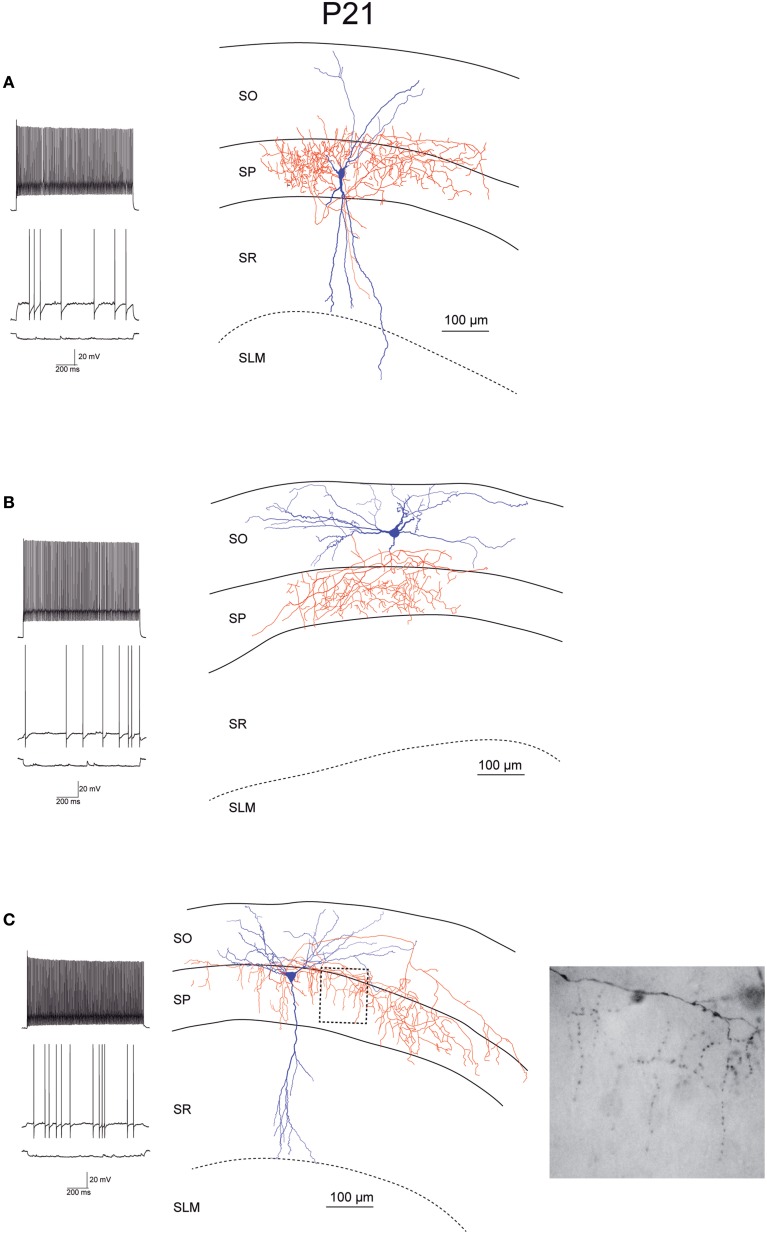
**Morphology and firing pattern of GluN2D-EGFP-positive cells in P21 transgenic mice. (A–C)** Reconstruction of three EGFP-GluN2D-positive cells (P21) with voltage responses to hyperpolarizing and depolarizing current injections. All three cells display fast spiking firing patterns. The axonal arbors of the upper two cells **(A,B)** are consistent with basket cells while the lower cell **(C)** resembles an axo-axonic cell. The image of part of the axon in the stratum pyramidale of the lower cell (see dashed box in reconstruction) shows vertical rows of boutons (parallel to axon initial segments), typical for axo-axonic cells. SO, Stratum oriens; SP, stratum pyramidale; SR, stratum radiatum; SLM, stratum lacunosum-moleculare.

While recording, we filled EGFP-GluN2D-positive cells with biocytin to obtain information about their morphology. The cell body of most filled neurons was located in the stratum pyramidale and the stratum oriens. Only few cells were located in the stratum radiatum. The dendritic and axonal arbor of EGFP-GluN2D-positive cells was diverse as expected from the different interneuronal marker proteins that they expressed. Dendrites of most cells were spine-free, with only some cells exhibiting a few spine-like protrusions. Axons usually had many boutons. Many filled cells could not be classified because they were stained too weakly or the axon was severed. Thirty two cells could be classified based on the localization of their dendritic and axonal arbor (Freund and Buzsaki, 1996; Klausberger and Somogyi, 2008). The majority had the morphology of soma targeting interneurons with basal and apical dendritic arbors in stratum oriens and stratum radiatum, respectively, and the main part of the axonal arbor in stratum pyramidale. Fifty percent of these cells resembled classical “pyramidal-shaped basket cells.” Most of them had the cell body in the stratum pyramidale, few in the lower stratum oriens. The dendritic arbor was reminiscent of that of pyramidal cells (i.e., a prominent dendrite that runs toward the stratum radiatum where it arborizes and a shorter dendritic arbor in the stratum oriens) (Figures 3–5) (Freund and Buzsaki, 1996). Most of the remaining interneurons with the main portion of the axon arbor in the stratum pyramidale (i.e., presumptive basket cells) had the cell body also in the stratum pyramidale, but had dendritic arbors different from that of pyramidal-shaped basket cells (e.g., little or no dendrites in stratum radiatum). Some neurons in slices of young mice (P5 and P9) had the appearance of immature basket cells with shorter dendritic arbors (Figures 3, 4). Four neurons resembled axo-axonic (chandelier) cells based on their axonal arbor in stratum pyramidale and lower stratum oriens with vertical rows of boutons (parallel to axon initial segments of pyramidal cells, Figures 3–5). Two of these cells had a rather immature appearance. Finally, two immature neurons in slices of P3 mice might be trilaminar and bistratified cells (or immature basket cells). A classification of biocytin-filled immature neurons based on morphology cannot be done with absolute certainty (Figures 3, 4).

Nevertheless, the morphology of many EGFP-GluN2D-positive cells corresponds with that of neurons identified based on the expression of interneuron marker proteins (in particular basket and axo-axonic morphologies are in accordance with the morphology of cells expressing PV). Although many EGFP-GluN2D-positive cells expressed SOM, we did not find a neuron with a classical O-LM morphology. There were several cells with cell body and horizontal dendritic arbor in stratum oriens (both features are indicative of O-LM cells) and axons directed toward stratum radiatum. However, the severed axon prevented a clear allocation to the class of O-LM cells (see Table 2 for anatomical properties and interneuron classification).

**Table 2:** **Anatomical properties of EGFP-GluN2D-positive cells**.

**PERCENTAGE OF NEURONS WITH LOCALIZATION OF CELL BODY IN**					
Stratum oriens	25%
Stratum pyramidale	68%
Stratum radiatum	7%
**PERCENTAGE OF NEURONS WITH LOCALIZATION OF MAIN PORTION**					
**OF AXON ARBOR IN**					
Stratum oriens	10%
Stratum pyramidale	84%
Stratum radiatum	6%
**CELL TYPE (% OF 32 NEURONS THAT COULD BE CLASSIFIED WITH**					
**SOME CERTAINTY)**					
Basket cells	69%
Immature basket cells	13%
Axo-axonic cells (chandelier)	13%
Trilaminar or bistratified cells	6%

### NMDAR-mediated synaptic currents in EGFP-GluN2D-positive cells

**Figure 6:**
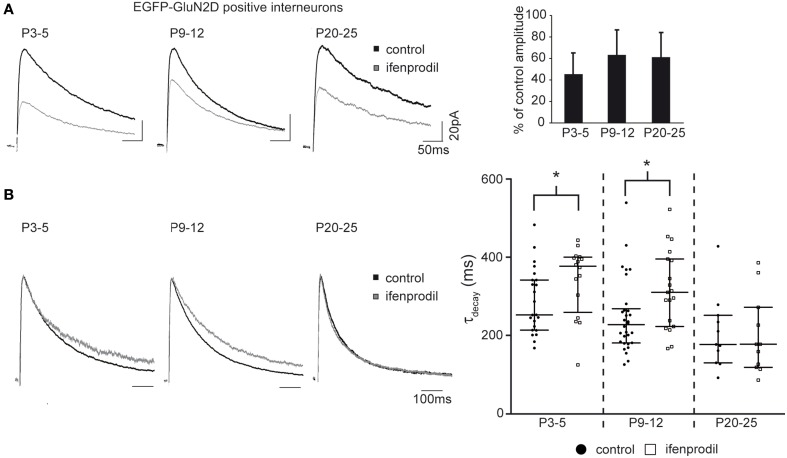
**Effects of ifenprodil on NMDAR-mediated EPSCs in GluN2D-EGFP-positive cells. (A)** Example traces of NMDAR-mediated EPSCs before and after application of the GluN2B specific antagonist ifenprodil. EPSCs were recorded at a holding potential of +40 mV in CA1 GluN2D-EGFP-positive interneurons in P3-5, P9-12, and P20-25 mice. The quantification of the remaining current after ifenprodil application shows that NMDARs containing other subunits than GluN2B contribute to synaptic currents at all three ages. **(B)** Example traces of NMDAR-mediated EPSCs before and after ifenprodil application normalized to the peak. The quantification of EPSC τ_decay_ shows that NMDARs with slower decay kinetics (i.e., GluN2D) than that of diheteromeric GluN2B-containing contributed to synaptic currents in GluN2D-EGFP-positive cells in P3-5 and P9-12 mice. The age-dependent decrease of τ_decay_ is consistent with an up-regulation of the “fast” GluN2A subunit. ^*^p < 0.05.

Next we asked if GluN2D receptors contribute to synaptic NMDAR-mediated currents. To this end, EGFP-GluN2D-positive CA1 interneurons were held at a membrane-potential of +40 mV and NMDAR-mediated EPSCs were evoked by Schaffer collateral/commissural fiber stimulation while blocking AMPA receptor- and GABA receptor-mediated currents. GluN2B is the most abundant subunit in hippocampal neurons during the first two postnatal weeks (Monyer et al., 1994). Therefore, to investigate if GluN2D-containing NMDARs mediate synaptic currents, we blocked GluN2B-containing NMDARs with 10 μM of the specific antagonist ifenprodil. This should result in an 80–90% block of dihetromeric GluN1/GluN2B receptors, a negligible block of diheteromeric NMDARs containing any other of the three subunits and most likely a moderate block of triheteromeric GluN2B-containing NMDARs (Mott et al., 1998; Hatton and Paoletti, 2005). NMDAR-mediated current amplitudes were significantly reduced in the presence of ifenprodil to approximately 50% in fluorescent interneurons of all three age groups (Figure 6A, Table 3) consistent with the presence of subunits other than GluN2B in the synapse of EGFP-GluN2D-positive CA1 interneurons. Deactivation time constants (τ_decay_) of NMDARs comprising different subunits vary considerably (GluN1/GluN2A 4-5 times faster than GluN1/GluN2B; GluN1/GluN2C similar to GluN1/GluN2B; GluN1/GluN2D 6–10 times slower than GluN1/GluN2B) (Monyer et al., 1994; Vicini et al., 1998). Thus, by comparing the τ _decay_ of NMDAR-mediated EPSCs before and after ifenprodil, we could infer the composition of unblocked NMDARs: if EPSCs τ_decay_ decreases, EPSCs should be mediated at least in part by fast GluN2A-containing NMDARs, if τ_decay_ increases, slower GluN2D-containing NMDARs should be involved. Interestingly, ifenprodil significantly increased τ_decay_ of NMDAR-mediated currents in P3-5 and P9-12 mice, indicating that GluN2D subunits contribute to synaptic NMDAR-mediated currents in EGFP-GluN2D-positive CA1 interneurons. In contrast, ifenprodil did not change τ_decay_ in P20-25 animals. τ_decay_ decreased with development from 253 to 177 ms most likely due to the increase in the contribution of fast GluN2A-containing NMDARs (Figure 6B, Table 3).

**Table 3:** **NMDAR-mediated currents**.

	**P3-5**	**P9-11**	**P20-25**
**EGFP-GLUN2D-POS. CELLS**			
Remaining current in the presence of ifenprodil (%)	45 ± 20 *n* = 14	63 ± 24 *n* = 9	60 ± 24 *n* = 5
τ_decay_ (ms)	253 [214–342] *n* = 23	227 [181–269] *n* = 30	177 [129–252] *n* = 11
τ_decay_ + ifenprodil (ms)	378 [260–401] *n* = 16^*^	311 [223–397] *n* = 19^*^	177 [118–272] *n* = 11
**PYRAMIDAL CELLS**			
Remaining current in the presence of ifenprodil (%)	32 ± 16 *n* = 12	47 ± 21 *n* = 8	72 ± 21 *n* = 10
τ_decay_ (ms)	312 [246-339] *n* = 26	254 [221–268] *n* = 23	241 [217–273] *n* = 15
τ_decay_ + ifenprodil (ms)	361 [271–601] *n* = 43^*^	215 [180–227] *n* = 22^**^	187 [168–219] *n* = 15^**^
**PV-POSITIVE CELLS**			
Remaining current in the presence of ifenprodil (%)		49 ± 17 *n* = 6	
τ_decay_ (ms)		230 [179–296] *n* = 31	
τ_decay_ + ifenprodil (ms)		268 [211–356] *n* = 40^*^	
***GRIN2D^−/−^* INTERNEURON**			
Remaining current in the presence of ifenprodil (%)	25 ± 20 *n* = 13		
τ_decay_ (ms)	323 [253–349] *n* = 21		
τ_decay_ + ifenprodil (ms)	303 [224–376] *n* = 14		
***GRIN2D*^−/−^ PYRAMIDAL CELLS**			
Remaining current in the presence of ifenprodil (%)	37 ± 17 *n* = 17		
τ_decay_ (ms)	361 [302–398] *n* = 20		
τ_decay_ + ifenprodil (ms)	267 [230–307] *n* = 11^**^		

Mean ± SD are shown for the percentage of remaining current in the presence of ifenprodil and median ± interquartile range [IQR]) for the decay time constants (τ_decay_). Statistical differences between the τ_decay_ in the absence and presence of ifenprodil were examined by Mann–Whitney U-test (

* p < 0.05,

** *p < 0.01)*.

### NMDAR-mediated synaptic currents in pyramidal cells

**Figure 7:**
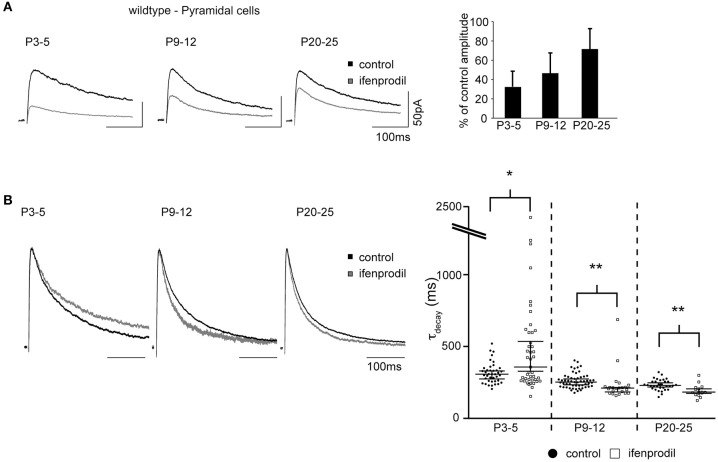
**Effects of ifenprodil on NMDAR-mediated EPSCs in pyramidal cells of wildtype mice. (A)** Example traces of NMDAR-mediated EPSCs before and after application of ifenprodil. EPSCs were recorded in CA1 pyramidal cells in P3-5, P9-12, and P20-25 wildtype mice. The quantification of the remaining current after ifenprodil application shows that NMDARs containing other subunits than GluN2B contribute to synaptic currents at all three ages. The decrease of blocking efficiency speaks for a developmental reduction in the proportional contribution of diheteromeric GluN2B-containing NMDARs. **(B)** Example traces of NMDAR-mediated EPSCs before and after ifenprodil application normalized to the peak. The quantification of EPSC τ_decay_ shows that NMDARs with slower decay kinetics than that of diheteromeric GluN2B-containing NMDARs contribute to synaptic currents in pyramidal cells in P3-5 mice. In contrast, ifenprodil decreases τ_decay_ in P9-12 and P20-25 mice, indicating that GluN2A-containing NMDARs contribute to synaptic currents. ^*^p < 0.05, ^**^p < 0.01.

We did not detect fluorescence in pyramidal cells in EGFP-GluN2D mice. However, several studies indicated that GluN2D is expressed in CA1 pyramidal and granule cells especially of young mice (Scherzer et al., 1998; Kirson et al., 1999; Hrabetova et al., 2000; Lozovaya et al., 2004; Harney et al., 2008). It is possible that EGFP-GluN2D expression is very faint in pyramidal cells of transgenic mice, or that all fusion-protein is transported into dendrites. In both cases pyramidal cells would appear non-fluorescent in spite of EGFP-GluN2D expression. On the other hand, BAC-transgenic mice sometimes display false-negative expression (Meyer et al., 2002; von Engelhardt et al., 2007). Hence we cannot take recourse to EGFP-GluN2D mice to help us identify pyramidal cells that might potentially express GluN2D. We used wildtype mice to investigate if GluN2D-containing NMDARs contribute to synaptic currents in pyramidal cells. The τ_decay_ decreased with development from 312 ms in P3-5 mice to 241 ms in P20-25 mice, consistent with the developmental upregulation of GluN2A. As expected, there was also a developmental decrease in the blocking efficiency of ifenprodil (Figure 7A, Table 3). Ifenprodil significantly increased the τ_decay_ in P3-5 mice, while there was a significant decrease in P9-11 and P20-25 mice (Figure 7B, Table 3), providing indirect evidence that slow GluN2D-containing NMDARs may contribute to synaptic currents in young mice.

### NMDAR-mediated synaptic currents in PV-positive cells

**Figure 8:**
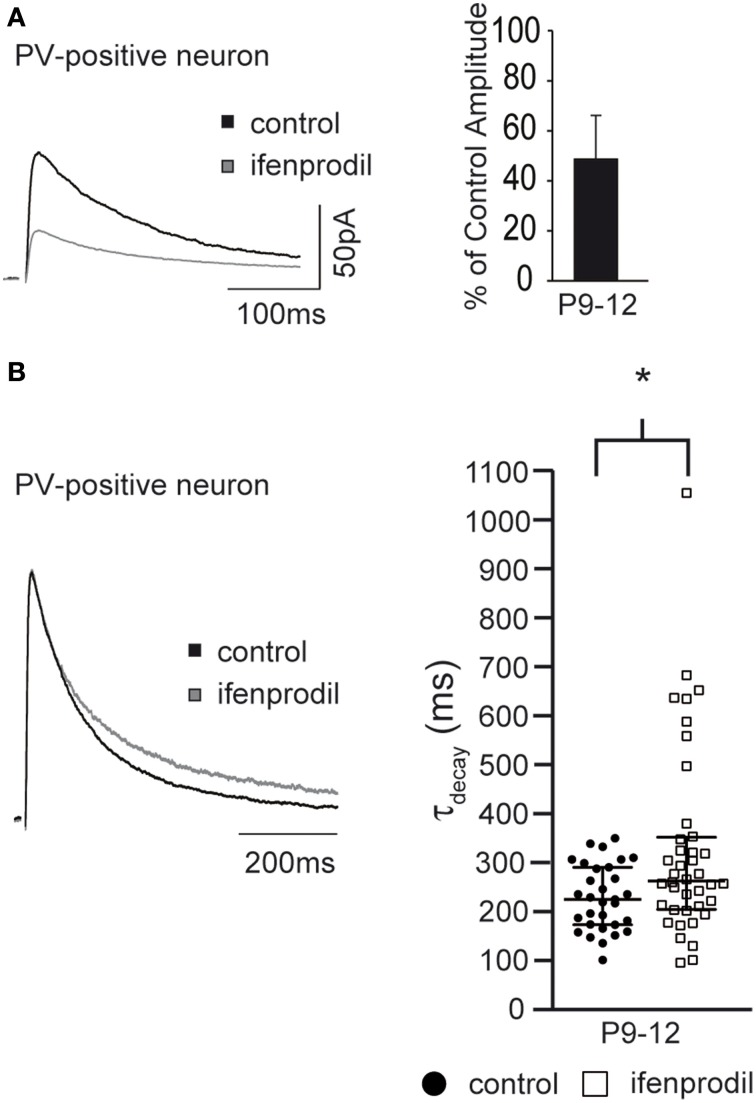
**Effects of ifenprodil on NMDAR-mediated EPSCs in P9-12 PV-EGFP-positive cells. (A)** Example traces of NMDAR-mediated EPSCs before and after application of ifenprodil. EPSCs were recorded in CA1 PV-EGFP interneurons of P9-12 mice. The quantification of the remaining current after ifenprodil application shows that NMDARs containing other subunits than GluN2B contribute to synaptic currents. **(B)** Example traces of NMDAR-mediated EPSCs before and after ifenprodil application normalized to the peak. The quantification of EPSC τ_decay_ shows that NMDARs with slower decay kinetics than that of diheteromeric GluN2B-containing NMDARs contribute to synaptic currents in PV-EGFP interneurons in P9-12 mice. ^*^p < 0.05.

EGFP-GluN2D expression in transgenic mice might result in a non-physiological expression of tagged GluN2D in synapses of fluorescent cells. Double labeling experiments showed that all hippocampal PV-expressing interneurons co-expressed EGFP-GluN2D. To corroborate our results obtained from EGFP-GluN2D mice, we investigated PV-positive cells that do not express EGFP-GluN2D. To this end, we took advantage of a transgenic mouse line in which PV-positive cells are labeled by EGFP (PV-EGFP mice) (Meyer et al., 2002). We could not analyze PV-positive cells in P3-5 mice, since there were no green cells at this age consistent with the appearance of PV expression only during the 2nd postnatal week (Nitsch et al., 1990). In PV-positive cells of P9-12 PV-EGFP mice, ifenprodil application resulted in a 50% reduction in NMDAR-mediated EPSC amplitude (Figure 8A, Table 3). Importantly, ifenprodil significantly increased τ_decay_ of NMDAR-mediated EPSCs in EGFP-positive interneurons in the CA1 region (Figure 8B, Table 3). These results demonstrate that NMDAR subunits with decay kinetics slower than those of GluN1/GluN2B diheteromeric NMDARs (i.e., GluN2D containing NMDARs) are present in the synapse of PV-positive interneurons.

### NMDAR-mediated synaptic currents in *grin2d^−/−^* mice

**Figure 9:**
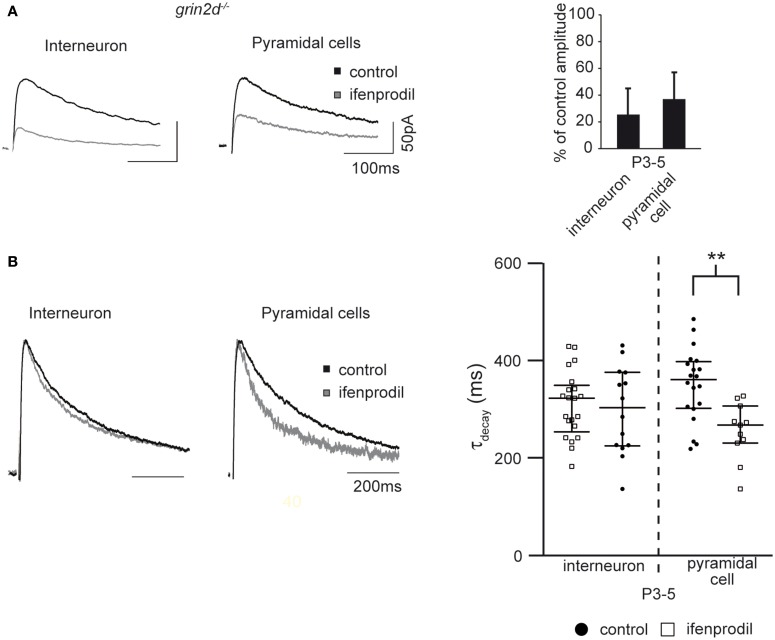
**Effects of ifenprodil on NMDAR-mediated EPSCs in pyramidal cells and stratum oriens interneurons in P3-5 grin2d*^−/−^* mice**. **(A)** Example traces of NMDAR-mediated EPSCs before and after application of the GluN2B specific antagonist ifenprodil. EPSCs were recorded in CA1 stratum oriens interneurons and CA1 pyramidal cells of P3-5 grin2d^−/−^ mice. The quantification of the remaining current after ifenprodil application is shown on the right. **(B)** Example traces of NMDAR-mediated EPSCs before and after ifenprodil application normalized to the peak. The quantification shows that ifenprodil does not alter τ_decay_ of EPSC in interneurons and decreases τ_decay_ of EPSC in pyramidal cells, suggesting that slow GluN2D subunits do not contribute to synaptic NMDARs in P3-5 grin2d^−/−^ mice, in contrast to their contribution in EGFP-GluN2D, PV-EGFP, and wildtype mice. ^**^p < 0.01.

The ifenprodil-mediated increase in τ_decay_ indicates that NMDAR subunits with slower kinetics than GluN2B subunits are present in the synapse of interneurons and pyramidal cells of young mice. If GluN2D subunits contribute to synaptic currents in hippocampal interneurons and pyramidal cells, we would expect that the ifenprodil-mediated increase in τ_decay_ is absent in mice lacking the GluN2D subunit (i.e., *grin2d^−/−^* mice). Since the increase in τ_decay_ was most pronounced in P3-5 mice, we analyzed NMDAR-mediated EPSCs in lower stratum oriens interneurons and pyramidal cells of P3-5 *grin2d^−/−^* mice. Ifenprodil significantly blocked NMDAR-mediated EPSCs in both cell types (Figure 9A, Table 3). In fact, ifenprodil was more efficient in blocking NMDAR-mediated EPSCs in interneurons of *grin2d^−/−^* than in interneurons of EGFP-GluN2D mice (remaining current 25 vs. 45 %). Considering that 10 μM ifenprodil blocks 80–90% of diheteromeric GluN2B-containing NMDARs, these results indicate that there are almost purely GluN1/GluN2B heteromeric NMDARs in interneuron synapses of *grin2d^−/−^* mice. Consistently, ifenprodil did not affect τ_decay_ of NMDAR-mediated EPSCs in interneurons (Figure 9B, Table 3), in contrast to the ifenprodil mediated τ_decay_ increase in EGFP-GluN2D-positive and in PV-positive interneurons. Ifenprodil decreased the τ_decay_ of NMDAR-mediated EPSCs in pyramidal cells in *grin2d^−/−^* mice (Figure 9B, Table 3), in stark contrast to the increase in wildtype mice. The fact that NMDAR-mediated EPSCs decayed even faster after blocking GluN2B-containing NMDARs indicates that fast GluN2A subunits are already expressed in synapses of pyramidal cells at an age of P3-5 old mice. In conclusion, these results are consistent with the loss of synaptic GluN2D-containing NMDARs in interneurons and pyramidal cells of *grin2d^−/−^* mice.

## Discussion

We generated BAC transgenic mice that express the fusion protein EGFP-GluN2D under the control of the GluN2D promoter with the aim to investigate the cellular identity of GluN2D-expressing cells in the hippocampus and to address the question of whether GluN2D-containing NMDARs mediate synaptic currents.

EGFP-GluN2D-positive cells had an interneuron identity as indicated by the localization of their cell body, electrophysiological properties, and neurite feature/arborization (Freund and Buzsaki, 1996; McBain and Fisahn, 2001; Klausberger and Somogyi, 2008). This is in accordance with a previous investigation that showed GluN2D mRNA expression in interneurons in the hippocampus of adult rats (Standaert et al., 1996). The lack of marker protein expression as well as immature anatomy and electrophysiological properties precludes an unequivocal classification of EGFP-GluN2D-positive interneurons in young mice. Anatomical and electrophysiological experiments showed that EGFP-GluN2D was expressed mainly in fast-spiking basket and axo-axonic PV-positive interneurons in the hippocampus of the adult mouse. PV expression commences in hippocampal interneurons during the 2nd postnatal week (Nitsch et al., 1990). Hence, many EGFP-GluN2D-positive cells that did not express PV in P9-12 old brains were most likely interneurons in which the expression of this protein had not started yet. Electrophysiological and anatomical characteristics of many EGFP-GluN2D-positive cells in young brains supported this hypothesis. However, EGFP-GluN2D was not only expressed in PV- but also in SOM-, CR, and CB-positive interneurons. A subpopulation of EGFP-GluN2D-positive cells must co-express PV and SOM, given that the summation of the percentage of co-localization was higher than 100%. Co-expression of PV and SOM in hippocampal interneurons was reported in previous studies (Dun et al., 1994; Klausberger et al., 2004). The fact that most PV- and more than half of the SOM-positive cells expressed EGFP-GluN2D is consistent with findings of Standaert and colleagues who performed double-labeling *in situ* hybridization experiments on adult brains using a ^35^S-labeled GluN2D probe and digoxigenin-labeled probes directed against GAD67, PV or SOM. The authors found co-labeling for GluN2D and the three interneuronal markers (Standaert et al., 1996).

Several studies indicated that hippocampal granule cells and pyramidal cells of young mice express GluN2D (Scherzer et al., 1998; Kirson et al., 1999; Hrabetova et al., 2000; Lozovaya et al., 2004; Harney et al., 2008). Kirson and colleagues showed by single cell RT-PCR that 50% of pyramidal cells express GluN2D mRNA during the 1st postnatal week (Kirson et al., 1999). Consistently, our electrophysiological recordings also indicated that GluN2D is expressed in pyramidal cells of P3-5 mice. The lack of EGFP-GluN2D expression in pyramidal cells of young mice might be explained by false-negative protein expression a frequent problem of BAC-transgenic mice (Meyer et al., 2002; von Engelhardt et al., 2007). Thus, it is possible that pyramidal neurons expressing GluN2D in newborn mice were not labeled by EGFP-GluN2D. In addition, EGFP-GluN2D-positive pyramidal neurons might appear un-labeled if most of the fusion protein is transported to dendrites and synapses such that the cell body compartment does not fluoresce.

Electrophysiological analysis of EGFP-GluN2D, PV-EGFP, wildtype and *grin2d^−/−^* mice showed that GluN2D-containing NMDARs mediate synaptic currents in hippocampal interneurons of P3-5 and P9-12 mice and CA1 pyramidal neurons of P3-5 mice. The presence of synaptic GluN2D-containing NMDARs in hippocampal interneurons of EGFP-GluN2D and PV-EGFP and CA1 pyramidal cells of P3-5 wildtype mice could be deduced from the ifenprodil-mediated increase of NMDAR-mediated EPSC τ_decay_. On the contrary, τ_decay_ did not change (interneurons) or decreased (pyramidal cells) in the presence of ifenprodil in P3-5 *grin2d^−/−^* mice. Ten micrometer ifenprodil blocked approximately 50% of NMDAR-mediated currents in EGFP-GluN2D and PV-EGFP mice but 75% in *grin2d^−/−^* mice, indicating that a substantial proportion of synaptic receptors comprise GluN2D-containing NMDARs in hippocampal interneurons of newborn mice.

NMDAR subunit composition changes dramatically with development. Thus, GluN2A expression increases in the entire brain and GluN2C in the cerebellum whereas GluN2B and GluN2D expression decreases (Monyer et al., 1994). Consistent with an up-regulation in the expression of fast GluN2A subunits, we observed a developmental decrease in the τ_decay_ of NDMA receptor-mediated EPSCs in hippocampal interneurons and pyramidal cells. A weak GluN2A mRNA signal was seen in *in situ* hybridization experiments in hippocampal neurons of P7 mice (Monyer et al., 1994). In fact, the ifenprodil-mediated decrease in τ_decay_ in pyramidal cells of P3-5 *grin2d^−/−^* mice indicates that GluN2A-containing NMDARs are already present at this early stage of development. Ifenprodil did not change the τ_decay_ in EGFP-GluN2D expressing interneurons of P20-25 old mice, although it decreased the amplitude of NMDAR-mediated currents to approximately 50%. This finding indicates that synaptic NMDARs comprise a mix of fast GluN2A, intermediate GluN2B and slow GluN2D subunits (be it in di- or triheteromeric receptors). The expression of only diheteromeric GluN1/GluN2A and GluN1/GluN2B receptors is unlikely since τ_decay_ would then decrease in the presence of ifenprodil. The expression of only triheteromeric GluN1/GluN2A/GluN2B is one possibility. However, the presence of EGFP-GluN2D fluorescence speaks for the persistent presence of GluN2D in PV- and SOM-positive interneurons of older mice.

We could not estimate the exact contribution of different subunits to synaptic NMDARs, since 10 μM ifenprodil blocks not only diheteromeric but also triheteromeric GluN2B-containing receptors to some extent (Hatton and Paoletti, 2005; Tovar et al., 2013). In addition, we have no information about their stoichiometry, i.e., we cannot infer from these experiments if synaptic GluN2D subunits form diheteromeric GluN1/GluN2D or triheteromeric GluN1/GluN2D/GluNX NMDARs. In fact, GluN2D can be contained in functional triheteromeric GluN1/GluN2D/GluNA receptors in *Xenopus* oocytes (Cheffings and Colquhoun, 2000). In addition, single channel recordings (Pina-Crespo and Gibb, 2002; Brickley et al., 2003; Jones and Gibb, 2005) and the analysis of NMDAR composition in protein preparations (Dunah et al., 1998) provided indication for the existence of triheteromeric GluN1/GluN2D/GluNA and GluN1/GluN2D/GluNB receptors in cerebellar Golgi cells, hippocampal granule cells and substantia nigra neurons in rat brains.

Previous reports showed that GluN2D-containing di- and triheteromeric NMDARs are present at extrasynaptic sites but are absent from synapses of different cell types indicating that GluN2D might exert a function exclusively extrasynaptically (Momiyama et al., 1996; Momiyama, 2000; Misra et al., 2000a,b; Brickley et al., 2003; Lozovaya et al., 2004). However, several recent reports suggested that GluN2D might also mediate synaptic currents (Harney et al., 2006, 2008; Logan et al., 2007; Brothwell et al., 2008). Interestingly, granule cells synapses are devoid of GluN2D during baseline recording, but GluN2D-containing NMDARs move into the synapse upon induction of long-term potentiation (LTP) of NMDAR-mediated EPSCs (Harney et al., 2008). In contrast, induction of long-term depression (LTD) of NMDAR-mediated EPSC in hilar interneurons is associated with removal of synaptic GluN2D (Harney and Anwyl, 2012).

Little is known about the function of GluN2D-containing NMDARs. Slow NMDAR-mediated currents with a large charge transfer via GluN2D and GluN2B-containing receptors are preponderant in the young brain and might allow the integration of synaptic input over longer time periods. The developmental up-regulation of GluN2A expression speeds up NMDAR-mediated currents thereby increasing the precision of cell-to-cell communication. Of note, there are several other developmental changes that would affect the temporal and spatial integration of synaptic inputs in hippocampal fast spiking interneurons. Thus, the total dendritic length increases whereas the membrane time constant decreases with development (Doischer et al., 2008). Changes in the composition of NMDARs might also affect the induction of synaptic plasticity. The slow kinetics of GluN2D-containing NMDARs could increase the time window for coincidence detection during induction of synaptic LTP or LTD. Moreover, as suggested by Hrabetova et al. (2000), only little depolarization might be required during LTP or LTD induction in synapses that express GluN2D-containing NMDARs since they display a weak Mg^2+^-block. LTP or LTD of synaptic AMPA receptor-mediated EPSCs in interneurons including CA1 PV-positive basket and axo-axonic cells usually depends on the activation of Ca^2+^-permeable AMPA receptors (Laezza et al., 1999; Lamsa et al., 2007; Oren et al., 2009; Nissen et al., 2010), in contrast to NMDAR-dependent LTP in principal cells (Bliss and Collingridge, 1993). However, an NMDAR-dependent LTP of feedback excitation from CA1 pyramidal cells onto distal dendrites of PV-positive interneurons has been observed (Le Roux et al., 2013).

*Grin2d^−/−^* mice display behavioral deficits comprising reduced spontaneous locomotor activity and sensitivity to stress and alterations in monoaminergic function (Ikeda et al., 1995; Miyamoto et al., 2002). GluN2D mRNA expression is high in the embryonic and immature postnatal brain but nearly absent in the adult brain (Watanabe et al., 1992; Monyer et al., 1994). Therefore, it was hypothesized that the deficits of *grin2d^−/−^* mice result from the lack of GluN2D function during embryogenesis and brain development and not from GluN2D expression and function in the adult brain. In light of our findings, it should be further considered whether some of the reported deficits in *grin2d^−/−^* mice might result from the absence of GluN2D in PV- and SOM-positive hippocampal interneurons.

### Conflict of interest statement

The authors declare that the research was conducted in the absence of any commercial or financial relationships that could be construed as a potential conflict of interest.

## Supplementary material

Figure S1 **Generation of GluN2D-EGFP BAC transgenic mice. (A)** Schematic representation of the wildtype BAC containing the *grin2d* gene, the recombination cassette, and the modified BAC containing EGFP-GluN2D. The position of HindIII and NotI restriction sites is indicated. The PCR fragment used as a probe for the Southern blot is indicated as a bar. **(B)** Southern blot analysis of tail DNA isolated from wildtype and transgenic mice digested with HindIII to compare signal intensities of the wildtype (5 kb) and transgene (2.7 kb) band. wt, wildtype; tg1 and tg2, transgenic mouse line 1 and 2. Electrophysiological and anatomical experiments were performed with mice from the tg1 line.

Figure S2 ***In situ* hybridization experiments show that transgenic mice display a correct expression pattern for GluN2D-EGFP**. *In situ* hybridization experiments with probes against EGFP **(A)** and GluN2D **(B)** on sagittal brain slices of P14 and P21 old mice. The similarity of the *in situ* hybridization signal pattern for EGFP in transgenic mice and GluN2D in wildtype mice shows that EGFP-GluN2D is expressed in the correct brain regions. The comparison of the *in situ* hybridization signal for GluN2D in wildtype and EGFP-GluN2D mice indicates that GluN2D is expressed at higher levels in the transgenic mouse especially in midbrain and brainstem structures.
