# Supplementary figures and images for: GluN2D-containing NMDA receptors-mediate synaptic currents in hippocampal interneurons and pyramidal cells in juvenile mice
Source: Front Cell Neurosci. 2015 Mar 25;9:95. doi: 10.3389/fncel.2015.00095 (PMC4373385; doi:10.3389/fncel.2015.00095)

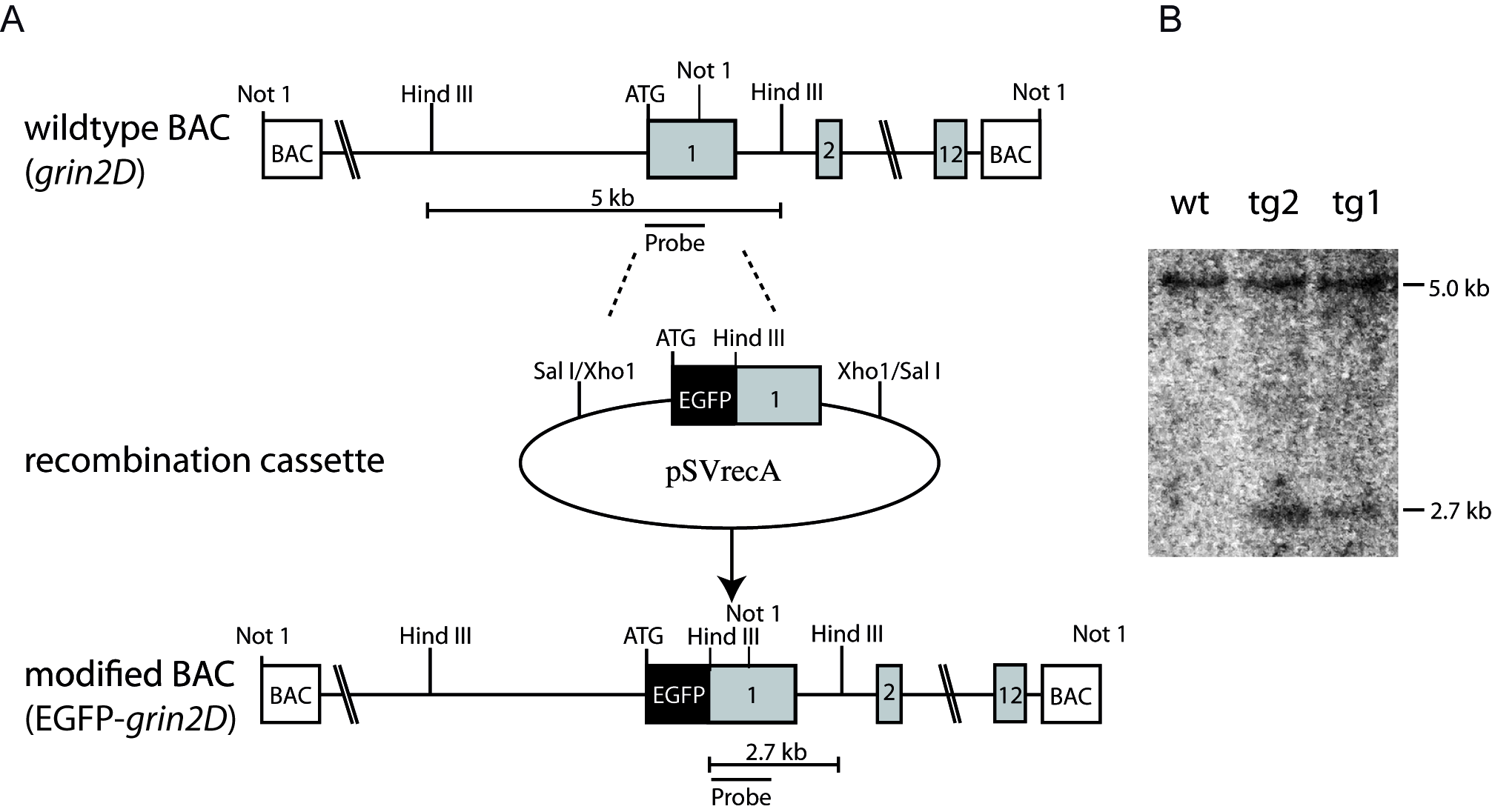

Supplement: Figure S1 — Generation of GluN2D-EGFP BAC transgenic mice. (A) Schematic representation of the wildtype BAC containing the grin2d gene, the recombination cassette, and the modified BAC containing EGFP-GluN2D. The position of HindIII and NotI restriction sites is indicated. The PCR fragment used as a probe for the Southern blot is indicated as a bar. (B) Southern blot analysis of tail DNA isolated from wildtype and transgenic mice digested with HindIII to compare signal intensities of the wildtype (5 kb) and transgene (2.7 kb) band. wt, wildtype; tg1 and tg2, transgenic mouse line 1 and 2. Electrophysiological and anatomical experiments were performed with mice from the tg1 line. [file Image1.TIF]

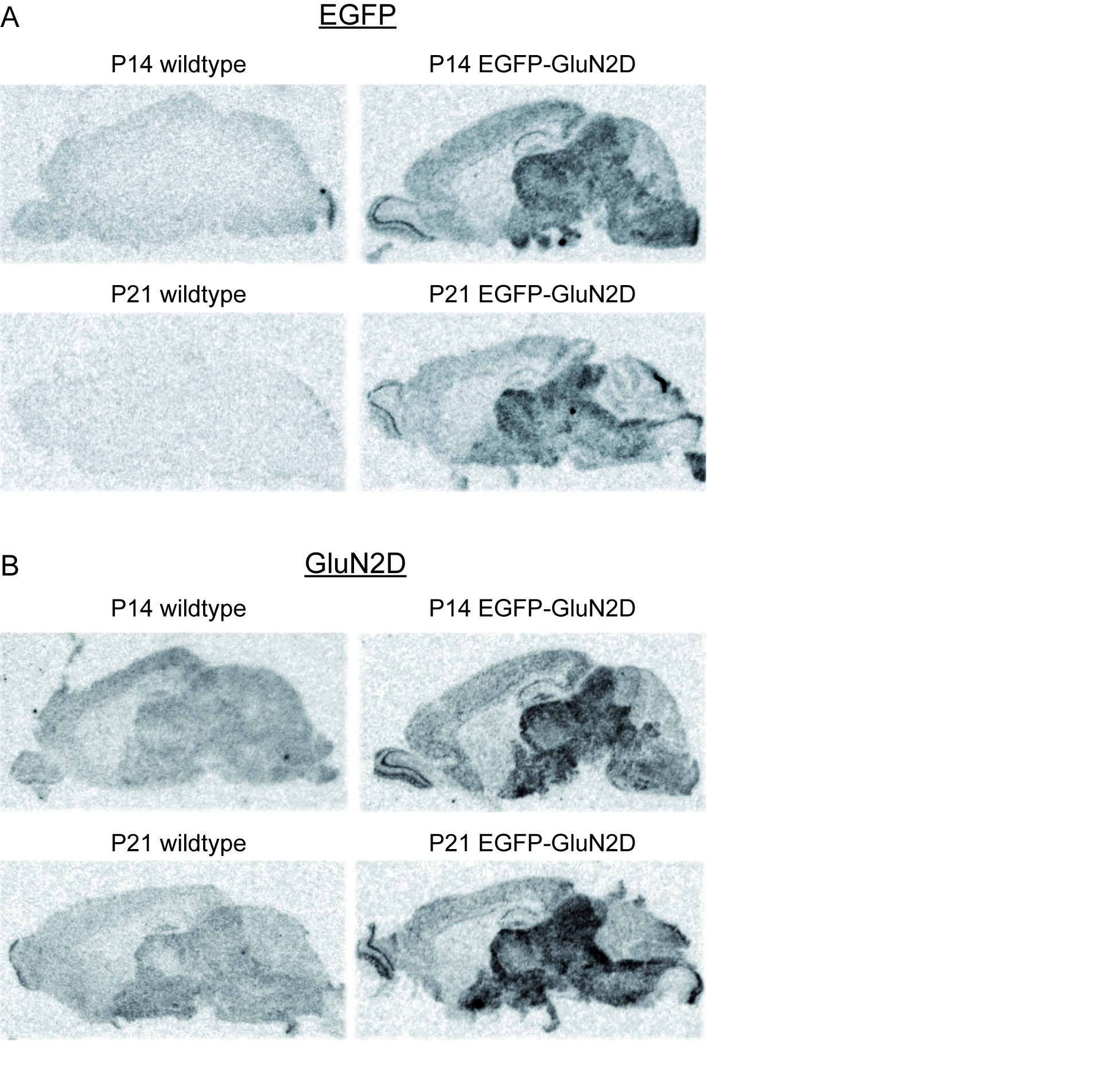

Supplement: Figure S2 — In situ hybridization experiments show that transgenic mice display a correct expression pattern for GluN2D-EGFP. In situ hybridization experiments with probes against EGFP (A) and GluN2D (B) on sagittal brain slices of P14 and P21 old mice. The similarity of the in situ hybridization signal pattern for EGFP in transgenic mice and GluN2D in wildtype mice shows that EGFP-GluN2D is expressed in the correct brain regions. The comparison of the in situ hybridization signal for GluN2D in wildtype and EGFP-GluN2D mice indicates that GluN2D is expressed at higher levels in the transgenic mouse especially in midbrain and brainstem structures. [file Image2.TIF]
